# Beyond COVID-19: Behavioral Drivers of Pandemic Prevention in Ghana’s Informal Urban Settlements

**DOI:** 10.1007/s11524-026-01106-x

**Published:** 2026-07-01

**Authors:** Richmond Amponsah, Marie Vaganay Miller, Aaron Lawson, Nigel McConnell

**Affiliations:** Belfast School of Architecture and the Built Environment, Ulster University, York Street, Belfast, Northern Ireland BT15 1ED UK

**Keywords:** COVID-19 pandemic, Prevention health behavior, Informal settlement, Sub-Saharan Africa, Structural equation modeling

## Abstract

The COVID-19 pandemic highlighted significant disparities in the uptake of precautionary health behaviors, particularly in informal settlements in sub-Saharan Africa (SSA), where access to essential infrastructure and socio-economic opportunities are limited. This study applied the information-motivation-behavioral (IMB) skills model of pandemic risk and prevention to explore the pathways through which COVID-19 information, personal and social motivation, and behavioral skills influenced the uptake of prevention behaviors in three informal settlements in Ghana. A survey of 496 participants was conducted and analyzed using structural equation modeling (SEM). Findings reveal a significant knowledge gap, with only 30.6% of participants answering all COVID-19 information questions accurately. Both personal (*β* = 0.209, *p* = 0.005) and social motivation (*β* = 0.178, *p* = 0.018) exhibited a direct significant relationship with preventive behaviors. While no direct effect was found between information and prevention behavior, behavioral skills fully mediated this relationship. Though focused on COVID-19, the findings have broader implications for strengthening pandemic/epidemic preparedness in informal settlements. Policy interventions should prioritize tailored health risk communication, motivation enhancement, and practical skills-building to empower vulnerable populations not just for current needs but to effectively respond to future health crises.

**Supplementary Information:**

The online version contains supplementary material available at https://doi.org/10.1007/s11524-026-01106-x.

## Introduction

The COVID-19 pandemic has profoundly impacted global public health, revealing significant variations in infection rates, disease severity, and preventive behaviors across different populations. While international public health organizations and governments emphasized non-pharmaceutical interventions such as wearing masks, maintaining hand hygiene, and practising social distancing, adherence to these behaviors remains inconsistent, particularly among informal urban settlements [1, 2]. This can be explained by the fact that these areas face several residential, occupational and social vulnerabilities, including overcrowding, inadequate water and sanitation, limited access to healthcare, and socio-economic inequalities [3]. For example, poor access to potable water and sanitation facilities, as well as a lack of space, renders preventive actions such as frequent handwashing and social distancing practically impossible in these settlements [4].

Understanding the determinants of pandemic preventive behaviors in these contexts is crucial for designing effective and tailored public health interventions that can better prepare communities for future health crises. This need is highlighted by the WHO warning that pandemics and outbreaks of infectious diseases are increasing more than ever, as recent decades have witnessed over five global pandemics, for example, Severe Acute Respiratory Syndrome Coronavirus (SARS-CoV), Middle East Respiratory Syndrome Coronavirus (MERS-CoV), Ebola, and, more recently, COVID-19 [5]. The COVID-19 pandemic has therefore reignited the necessity of building resilient urban communities, especially in informal urban settings where vulnerabilities are most acute [2]. A recent review has revealed a growing body of empirical studies on the behavioral determinants and response of COVID-19 in informal settlements [6]. The review identified multiple factors influencing preventive behaviors, including knowledge, attitudes, perceptions, and structural barriers. The authors noted that there is limited empirical research that critically examines the nuanced connections among knowledge, attitude, and practice. Most existing studies are mainly descriptive, offering only a snapshot of perception and behavior during the COVID-19 pandemic in informal urban settlements [6]. The authors explicitly called for future research applying a pandemic-based behavioral change model to unpack the complexities of the COVID-19 knowledge-attitude-practice relationship and the factors which mediate such links [6]. Health behavioral theories provide a systematic framework for analyzing and predicting determinants of and understanding health behaviors [7].

One such widely used theory is the information-motivation-behavioral (IMB) skills model, originally developed by Fisher and Fisher to predict and understand AIDS-risk behavior [8]. More recently, the authors have developed a pandemic-specific extension of the traditional model—the IMB skills model of pandemic risks and prevention to explicitly account for the specific challenges of emerging infectious diseases [9]. Over decades, the IMB skills model has proven effective in understanding and assessing the determinants of epilepsy management [10], osteoporosis self-management behavior [11], smoking cessation [12, 13], safe and risky sexual behaviors [14, 15], dietary behaviors [16, 17], and medication adherence for rare diseases (e.g., HIV/AIDS and diabetes) [18]. More recently, the IMB model has been applied to COVID-19 prevention behaviors among the general populations, predominantly in the Asian context [19–21]. For instance, in applying the model to COVID-19 behaviors among several islands of Indonesia, [21] found that both information and motivation positively influence individuals’ behavioral changes, with behavioral skills serving as a mediating factor in the relationships among information, motivation, and behavior. Similarly, Luo and colleagues introduced two additional constructs (perceived health stress risk and positive perceptions of interventions) into the model to predict COVID-19 health behaviors among Chinese people [20]. Results showed that information, motivation, behavioral skill, positive perceptions of interventions, and perceived health risks significantly influenced health behaviors. Accordingly, behavioral skills were the most significant predictor of health behaviors [20]. These findings, however, affirm that the IMB model is well established in understanding health behaviors.

Despite the growing body of IMB-COVID-19 research, to our knowledge, no known study has applied the IMB skills model of pandemic risk and prevention in informal urban settlements, particularly in sub-Saharan Africa (SSA). This gap is concerning for two main reasons. First, more than half of the region’s urban population resides in such settlements, where residents are disproportionately vulnerable to a range of public health risks [22]. Secondly, the importance of context in such studies must be emphasized, as lessons learned from broader populations or different regions where the IMB model has been validated may not be applicable to informal settlements. These settings (especially in SSA) face unique structural constraints and characteristics, including economic precarity, socio-cultural factors, overcrowding, limited access to infrastructure (e.g., water, sanitation), and a complex governance and political landscape. Consequently, these factors may alter the relationships among information, motivation, and behavioral skills and further influence how they engage with recommended behaviors. Therefore, we contend that the model’s predictive capacity is not uniform across contexts and populations. In this regard, extrapolating findings to informal settlement dwellers and other low-resource settings in SSA may be inappropriate for public health policy formulation, given the inherent uncertainties.

To address these gaps, this study applied the IMB skills model of pandemic risk and prevention (a mediation model) using structural equation modeling (SEM) to analyze quantitative survey data to investigate the factors influencing pandemic prevention behaviors in informal urban settlements in Ghana, specifically (1) to explore whether pandemic prevention information, pandemic prevention motivation (personal and social motivation), and pandemic prevention behavioral skills directly influence pandemic prevention behaviors, and (2) to examine whether pandemic prevention behavioral skills play a mediating role in the relationships between pandemic prevention information, pandemic prevention motivation (personal and social motivation), and pandemic prevention behaviors. This study is significant for several reasons. It offers empirical insights into how information, motivation, and behavioral skills shape pandemic behaviors in marginalized communities. By applying advanced analytical techniques, it deepens understanding of psychosocial drivers in marginalized contexts and contributes to both theory and practice. The findings can inform behaviorally grounded, context-specific strategies to enhance pandemic preparedness and the resilience of vulnerable populations.

## Theoretical Framework and Hypothesis Development

This study is guided by the IMB skills model of pandemic risk and prevention [9]. Unlike the original IMB model, the pandemic-specific extension model mainly accounts for significant conditions. These include rapidly evolving information, uncertainty about transmission dynamics, the role of misinformation, the need for adaptive behavioral skills as public health recommendations change, and the structural barriers that may influence IMB pathways in different contexts, such as marginalized communities. The model posits that three core determinants—pandemic information, pandemic motivation, and pandemic prevention behavioral skills—are the key determinants of pandemic prevention behaviors. Accordingly, it proposes that information (accurate, up-to-date knowledge about pandemics’ threat, how it spreads, its symptoms, and how it can be prevented) and motivation (personal attitudes and perceived social norms and support) work primarily through behavioral skills (objective skills and perceived self-efficacy) to influence behaviors, with behavioral skills acting as the most proximal determinant of action [8, 9]. Given the importance of personal and social factors in influencing health behaviors, we decompose pandemic prevention motivation to capture these dimensions independently. In line with the model’s assumptions, pandemic prevention information, personal motivation, and social motivation are allowed to covary. Based on these theoretical assumptions, the following hypotheses are proposed:

Predictors of pandemic prevention behavioral skills

**H1:** Pandemic prevention information is positively associated with pandemic prevention behavioral skills.

**H2:** Pandemic prevention personal motivation is positively associated with pandemic prevention behavioral skills.

**H3:** Pandemic prevention social motivation is positively associated with pandemic prevention behavioral skills.

Predictors of pandemic prevention behaviors

**H4:** Pandemic prevention information is positively associated with pandemic prevention behaviors.

**H5:** Pandemic prevention personal motivation is positively associated with pandemic prevention behaviors.

**H6:** Pandemic prevention social motivation is positively associated with pandemic prevention behaviors.

**H7:** Pandemic prevention behavioral skills are positively associated with pandemic prevention behaviors.

Mediation effect of pandemic prevention behavioral skills

**H8:** Pandemic prevention behavioral skills mediate the relationship between pandemic prevention information and pandemic prevention behaviors.

**H9:** Pandemic prevention behavioral skills mediate the relationship between pandemic prevention personal motivation and pandemic prevention behaviors.

**H10:** Pandemic prevention behavioral skills mediate the relationship between pandemic prevention social motivation and pandemic prevention behaviors.

## Materials and Methods

### Study Setting and Participant Recruitment

The study was conducted in the Greater Accra Region (GAR), the most populous region, with several informal settlement pockets [23]. Additionally, GAR was the epicenter of COVID-19 in Ghana, accounting for 56.8% of cases [24]. Three high-density, long-established settlements—Old Fadama, Nima, and Alogboshie—were selected for their large migrant populations, limited infrastructure and services, public health risks, and history of evictions [25]. Available data estimate the populations of the settlements are as follows: Old Fadama (100,000), Alogboshie (13,200), and Nima (80,843) [26–28]. The minimum required sample size of 383 for the combined population of the settlements was determined using the Cochran formula at the 95% confidence level [29]. To ensure adequate representation, the study was oversampled to 512 respondents. The sample was then proportionally distributed across the three settlements based on their population sizes. This sample is consistent with previous COVID-19 behavioral studies conducted in selected slum communities [30, 31]. We used a multi-pronged approach to participant recruitment. Data were collected between September and December 2024 using face-to-face surveys administered on tablets using the KoboCollect application. Trained enumerators who were also residents of the selected communities assisted with data collection. In each settlement, enumerators were assigned to different areas to ensure spatial coverage and reduce clustering. Within households, one participant was randomly selected if they met the inclusion criteria: aged 18 years or older, with at least 5 years of residency, and willing to provide consent. The 5-year minimum residency criterion ensured participants had sufficient exposure to local COVID-19 experiences and public health messaging.

### Measurement Instruments

To operationalize the theoretical model of the study, a structured survey questionnaire was developed, with survey items adapted from previous studies and contextualized. For details on the constructs, their respective measurement items, and internal consistency, see Table 1; detailed information on the measurement items, response coding, and descriptive statistics is provided in the Supplementary Material (Table S1). Pandemic prevention information was measured using ten items assessing participants’ objective knowledge of COVID-19 transmission modes, symptoms, and precautionary measures [20, 32]. Responses were coded as correct (1) or incorrect/“Don’t Know” (0), with total scores categorized into low (< 60%), moderate (60%–79%), and higher (80%–100%) knowledge levels using Bloom’s cut-off points [33]. Only 30.6% of participants answered all questions correctly. This construct was not included in the confirmatory factor analysis and was modeled as an observed categorical variable in the path analysis.

**Table 1:** Constructs and measurement items (*N* = 496)

Constructs	Items
Pandemic prevention information (PPI) (Cronbach’s *α* = 0.786)	PPI_1 = Could drinking unclean water make one contract COVID-19?
	PPI_2 = Are the primary transmission methods of COVID-19 by droplets and contact with an infected person?
	PPI_3 = Could drinking warm water, ginger, and lemon kill the COVID-19 virus?
	PPI_4 = In general, once infected with COVID-19, does it take 2–14 days for symptoms to show?
	PPI_5 = Could people with COVID-19 with no symptoms spread the virus to others?
	PPI_6 = Could taking antibiotics prevent COVID-19?
	PPI_7 = Is loss of smell or taste a symptom of COVID-19?
	PPI_8 = Is the elderly the only group at risk of getting COVID-19?
	PPI_9 = Could frequent handwashing prevent an individual from contracting COVID-19?
	PPI_10 = Could a nose/face mask protect one from contracting COVID-19?
Pandemic prevention personal motivation (PPPM) (Cronbach’s *α* = 0.902)	PPPM_1 = I thought COVID-19 was very contagious
	PPPM_2 = I was afraid of contracting COVID-19 in 2020 because I could die
	PPPM_3 = I know that getting sick with COVID-19 can be very severe
	PPPM_4 = I believe that taking the recommended preventive measures can significantly reduce my risk of COVID-19 infection
Pandemic prevention social motivation (PPSM) (Cronbach’s *α* = 0.833)	PPSM_1 = Following the appropriate recommended practices (e.g., a nose/face mask, washing of hands, applying hand sanitizer, etc.) could prevent those around me (family, friends and neighbors) from contracting COVID-19
	PPSM_2 = My family and neighbors were willing to support my health behaviors
	PPSM_3 = I believe that it is a shared responsibility within the community to collectively work toward preventing the spread of COVID-19
Pandemic prevention behavioral skills (PPBS) (Cronbach’s *α* = 0.864)	PPBS_1 = I actively paid attention to real-time COVID-19 information
	PPBS_2 = I could accurately identify and monitor my health for the common symptoms of COVID-19
	PPBS_3 = I felt confident in my ability to protect myself against COVID-19
	PPBS_4 = I could put on a face mask correctly, ensuring it covers my nose and mouth
Pandemic prevention behaviors (PPBH) (Cronbach’s *α* = 0.854)	PPBH_1 = Wash hands regularly with soap and running water
	PPBH_2 = Apply alcohol-based hand sanitizers
	PPBH_3 = Maintain a safe physical distance from people when outside (at least 1 m)
	PPBH_4 = Wear nose/face masks whenever I am in public places
	PPBH_5 = Cover my mouth and nose with a tissue or elbow when coughing or sneezing
	PPBH_6 = Avoid crowded areas

The remaining constructs were treated as latent variables. Pandemic prevention personal motivation, social motivation, and behavioral skills were each measured on a 5-point Likert scale (1 = strongly disagree to 5 = strongly agree). Personal motivation included four items, such as *“*I thought COVID-19 was very contagious,” adapted from [20, 31]. Social motivation involved three items (e.g., “My family and neighbours were willing to support my health behaviours”) drawn from [20, 34]. Behavioral skills were assessed through four items adapted from [20, 31] to evaluate participants’ ability and self-efficacy to perform COVID-19 prevention behaviors (e.g., “I could accurately identify and monitor my health for the common symptoms of COVID-19”). Pandemic prevention behaviors were assessed with six items measuring how often participants engaged in practices such as mask-wearing and handwashing during the peak of the pandemic, using a 5-point Likert scale (1 = never to 5 = always). Socio-demographic characteristics include gender, age, household size, marital status, educational attainment, religion, employment status, residential tenure, and monthly income.

### Analytical Procedures

We combined the Statistical Package for the Social Sciences (SPSS) IBM version 29.0.1.0, with the lavaan package in RStudio version 4.4.2. We employed SEM to analyze our data, as it differs from first-generation statistical methods in that it simultaneously examines relationships between exogenous and endogenous variables, accounts for confounding and methodological errors, and assesses both direct and indirect effects [35]. A two-step data analysis process was conducted. First, a confirmatory factor analysis (CFA), also known as the measurement model, was conducted for the latent variables and was validated using construct validity measures, including standardized factor loadings, average variance extracted, construct reliability, and multiple observed indicators. Second, a structural path analysis was estimated to test the hypothesized relationship among the constructs. We used weighted least squares means and variance adjusted (WLSMV) as the model estimation method, as it appears to perform well across various conditions, including small to large sample sizes and categorical or ordinal data (e.g., Likert-type scales) with non-normal distributions [36–38]. *p*-values < 0.05 were considered to be significant.

## Results

### Socio-demographic Characteristics of Participants

Of the 512 survey responses, 16 were excluded as they did not meet the study’s inclusion criteria and were incomplete. Out of the 496 participants, 56.5% were male and 43.5% were female, resulting in a somewhat balanced gender distribution. Most participants (59.2%) fell within the 25–44 age range, reflecting the demographics of informal settlements, where younger populations tend to predominate. A significant proportion (68.5%) of participants were employed, with many engaged in informal activities such as trading, vending, mechanics, 'okada' riding, phone sales, carpentry, and masonry. Income levels indicate that more than half (56.7%) of the participants earned GHS 1000 or less per month, while only 1.8% earned more than GHS 2500. The reliance on informal work and low incomes highlights economic vulnerability typical of low-resource settings. Most (58.1%) identified as Muslims, followed by Christians at 37.3%. Households average 4.21 people, and residents have lived in the community for about 19.23 years. Table 2 presents a comprehensive summary of the socio-demographic characteristics of the participants.

**Table 2:** Participants’ socio-demographic characteristics

**(*N* = 496) variables**	**Specification**	**Frequency**	**Percentage (%)**
**Gender**	Male	280	56.5
	Female	216	43.5
**Age (years)**	18–24	63	12.7
	25–34	142	28.6
	35–44	152	30.6
	45–54	73	14.7
	55–64	49	9.9
	65 and above	17	3.4
**Highest level of education**	No formal education	103	20.8
	Primary school	79	15.9
	Junior secondary/high school	111	22.4
	Senior secondary/high school	146	29.4
	Vocational/technical/commercial	15	3.0
	Tertiary	42	8.5
**Religion**	Christian	185	37.3
	Muslim	288	58.1
	Traditional	12	2.4
	No affiliation	8	1.6
	Others	3	0.6
**Marital status**	Single	182	36.7
	Married	268	54.0
	Divorced	19	3.8
	Widowed	23	4.6
	Co-living	4	0.8
**Residential status**	Owner	154	31.0
	Renter	307	61.9
	Free occupant	35	7.1
**Household size**	1–3	238	48.0
	4–6	191	38.5
	7 and above *Mean household size* = ***4.21* ± *3.53***	67	13.5
**Employment status**	Student	45	9.1
	Employed	340	68.5
	Unemployed	111	22.4
**Monthly income (GHS)**	1–500	98	19.8
	501–1000	183	36.9
	1001–1500	120	24.2
	1501–2000	65	13.1
	2001–2500	21	4.2
	2501 and above	9	1.8

Source: Field Survey (2024)

*Note*: the category “others” under religion comprises Atheist, Buddhist, and Rastafari

### Confirmatory Factor Analysis

The analysis begins with a CFA to validate the latent constructs. We used a range of incremental and absolute fit indices to assess the fitness of the measurement model, as different indices reflect distinct aspects of model fit [39, 40]. Results indicate a good fit: *χ*^2^/df = 2.639, *p*-value = 0.000; comparative fit index (CFI) = 0.984; Tucker-Lewis index (TLI) = 0.980; standardized root mean square residual (SRMR) = 0.041; and root mean square error of approximation (RMSEA) = 0.058. The CFA results, including standardized factor loadings, composite reliability (CR), average variance extracted (AVE), and descriptive statistics for the constructs, are presented in Table 3.

**Table 3:** Measurement properties of constructs, including reliability and validity indices (*N* = 496)

Constructs (total score range)	Std	SMC	Mean	SD	CR	AVE	Cronbach’s *α*
Pandemic prevention information (0–10) Knowledge score	-	-	7.20	2.440	-	-	0.786
Pandemic prevention personal motivation (4–20)			16.20	3.085	0.931	0.770	0.902
PPPM_1	0.862	0.744					
PPPM_2	0.891	0.794					
PPPM_3	0.917	0.841					
PPPM_4	0.840	0.706					
Pandemic prevention social motivation (3–15)			11.92	2.362	0.875	0.700	0.833
PPSM_1	0.849	0.721					
PPSM_2	0.818	0.669					
PPSM_3	0.843	0.710					
Pandemic prevention behavioral skills (4–20)			16.04	2.953	0.903	0.702	0.864
PPBS_1	0.857	0.734					
PPBS_2	0.878	0.770					
PPBS_3	0.772	0.597					
PPBS_4	0.841	0.708					
Pandemic prevention behaviors (6–30)			20.90	4.840	0.883	0.559	0.854
PPBH_1	0.736	0.542					
PPBH_2	0.854	0.730					
PPBH_3	0.667	0.445					
PPBH_4	0.764	0.583					
PPBH_5	0.738	0.544					
PPBH_6	0.714	0.509					

Note: Pandemic prevention information is modeled as a manifest ordinal variable derived from an aggregate index; therefore, factor loadings, AVE, and CR are not applicable. Scale reliability is represented by Cronbach’s alpha (*α* = 0.786)

*Std*, standardized factor loadings; *SMC*, squared multiple correlations; *CR*, composite reliability; *AVE*, average variance extracted

Construct validity measures how well a measurement scale represents the constructs it purports to assess. Validity analysis encompasses both convergent and discriminant validity assessments. The convergent validity of the constructs was estimated using AVE [41]. The AVE values were above the threshold of 0.50 [41]. Additionally, all alpha values were greater than 0.60. Therefore, the scales used in the present study demonstrate the necessary convergent validity. Discriminant validity was also assessed using the Fornell and Larcker criterion. According to the Fornell and Larcker criterion, discriminant validity is established when the square root of the AVE for a construct is greater than its correlation with other constructs. The values presented in this study (Table 4) denote that discriminant validity is established. However, the criterion proposed by Fornell and Larcker has been criticized in recent studies, prompting the introduction of a new method, the Heterotrait-Monotrait (HTMT) ratio. The assessment of HTMT in Table 5 revealed that all ratios were below the required limit of 0.85 [42], confirming satisfactory discriminant validity.

**Table 4:** Discriminant validity—Fornell-Larcker criterion

Latent variable	PPPM	PPSM	PPBS	PPBH
PPPM	***0.878***			
PPSM	0.661	***0.837***		
PPBS	0.548	0.539	***0.838***	
PPBH	0.474	0.463	0.469	***0.748***

*Note*: The diagonal values (in bold italics) indicate the square roots of the AVE, and the off-diagonal values indicate the correlations between the latent constructs

*PPPM*, pandemic prevention personal motivation; *PPSM*, pandemic prevention socialmotivation; *PPBS*, pandemic prevention behavioral skills; *PPBH*, pandemic preventionbehaviors

**Table 5:** Discriminant validity—Heterotrait-Monotrait ratio (HTMT)

Latent variable	PPPM	PPSM	PPBS	PPBH
PPPM				
PPSM	0.442			
PPBS	0.363	0.339		
PPBH	0.294	0.276	0.278	

Note: *PPPM*, pandemic prevention personal motivation; *PPSM*, pandemic prevention social motivation; *PPBS*, pandemic prevention behavioral skills; *PPBH*, pandemic prevention behaviors

### Path Analysis

**Fig. 1:**
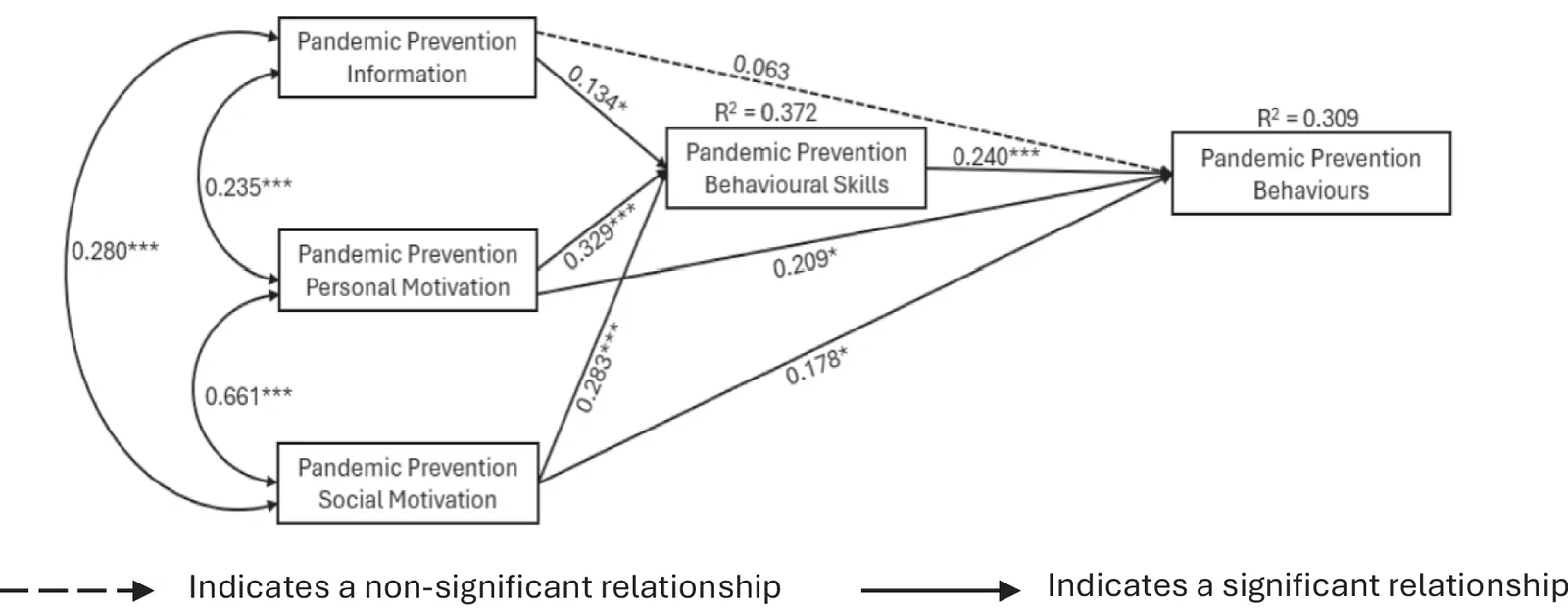
The final structural model of the IMB skills model of pandemic risk and prevention. Coefficients are standardized path coefficients. ***p < 0.001. *p < 0.05

To assess whether and how the variables in the IMB skills model of pandemic risk and prevention affect COVID-19 preventive behavior, a mediation analysis was conducted. The model showed a good fit (*χ*^2^/df = 2.55, *p*-value = 0.000; CFI = 0.983; TLI = 0.979; SRMR = 0.041, and RMSEA = 0.053). Results in Fig. 1 show that the paths linking personal motivation and behavioral skills (*p* < 0.001), social motivation and behavioral skills (*p* < 0.001), and behavioral skills and prevention behavior (*p* < 0.001) are all positive and statistically significant, indicating that both personal and social motivation strongly influence the development of behavioral skills, which in turn translate into prevention health behaviors. Similarly, the paths from personal motivation (*β* = 0.209**,** *p* = 0.005) and social motivation (*β* = 0.178**,** *p* = 0.018) to prevention behavior were significant, suggesting that motivation exerts a direct effect on behavior in addition to its indirect effect through behavioral skills.

Accordingly, the results suggest that the effect of information on prevention behavior is fully mediated by behavioral skills, as the direct path from information to behavior was not significant (*β* = 0.063, *p* = 0.198) (Fig. 1), whereas the indirect path via behavioral skills was significant (*β* = 0.032, *p* = 0.019) (see Table 6). For personal and social motivation, partial mediation was observed, as both the direct and indirect effects on behavior remained significant (*p* < 0.01). The results further indicate that information, personal motivation, and social motivation together explained 37.2% of the variation in behavioral skills, while the predictors explained 30.9% of the variation in prevention behavior. This demonstrates that these variables possess a moderate ability to explain behavioral skills and preventive behaviors. In addition, personal and social motivation were strongly correlated (*p* < 0.001), and both were positively associated with information (*p* < 0.001), indicating that individuals who were more informed also tended to be more motivated (personally and socially).

**Table 6:** Mediating effect of pandemic prevention behavioral skills

Path	Effect type	Std. (*β*)	std err	***z***-value	***p***-value	95% CI		Conclusion
						Lower bound	Upper bound	
PPI → PPBS → PPBH	Indirect	0.032	0.010	2.339	0.019**	0.004	0.044	Full mediation
PPPM → PPBS → PPBH	Indirect	0.079	0.020	3.357	0.001***	0.028	0.107	Partial mediation
PPSM → PPBS → PPBH	Indirect	0.068	0.020	2.925	0.003**	0.019	0.098	Partial mediation
PPI → PPBH	Total	0.095	0.037	1.873	0.061*			
PPPM → PPBH	Total	0.288	0.060	4.104	0.000***			
PPSM → PPBH	Total	0.246	0.064	3.327	0.001**			

Notes: *PPI*, pandemic prevention information; *PPPM*, pandemic prevention personal motivation; *PPSM*, pandemic prevention social motivation; *PPBS*, pandemic prevention behavioral skills; *PPBH*, pandemic prevention behaviors; *Std. (β)*, standardized estimate

***p-value < 0.001, **p-value < 0.05, *p-value < 0.10

## Discussion

In this study, we applied the IMB skills model of pandemic risk and prevention to explore whether and how information and knowledge, motivation (both personal and social), and behavioral skills influence COVID-19 pandemic preventive behaviors in informal urban settlements. In accordance with the IMB skills model, the findings from this study provide evidence supporting the hypothesis that personal motivation directly influences COVID-19 prevention behaviors. This finding underlines the critical role of individual-level attitudinal and cognitive factors, such as perceived threat, internal beliefs, and perceived benefits of interventions, in influencing adherence to and engagement in recommended health behaviors to prevent disease [9, 43, 44]. For instance, in this study, participants commonly agreed that COVID-19 is highly contagious, potentially severe, and that adhering to recommended COVID-19 preventive measures was crucial for minimizing their risk of exposure to and contracting the virus. This finding aligns with previous studies (see [20, 31, 45, 46]). For example, [45] found that the perceived severity posed by the threat of the virus increased fear arousal, which consequently played a significant role in influencing the adoption of COVID-19 prevention behaviors among Taiwanese individuals. Additionally, the recognition of perceived vulnerability to COVID-19 has been shown to motivate preventive behaviors among urban slum dwellers in Bangkok, Thailand [31]. Indeed, a person is likely to be highly motivated to adopt preventive practices if they view the threat of infection as serious and believe that adopting behavioral measures effectively reduces their health risk [44]. Therefore, interventions designed to promote pandemic-related health behaviors in low-resource settings and yield positive health outcomes should focus on enhancing personal motivation by reinforcing internal beliefs of people and perceived threats of the disease. This has vital implications for developing risk-tailored communication strategies and behavioral interventions that will strengthen individual risk perceptions and the effectiveness of preventive actions to improve adherence to appropriate public health measures.

Additionally, our results show that social motivation has a significant and direct impact on prevention behavior. This suggests that personal risk perceptions do not solely drive individuals’ behaviors in such contexts but are also influenced by their social environment and the expectations, support, and norms within their community. As the risk of infection extends from personal vulnerability to include potential impacts on close or significant others (e.g., family, friends, neighbors), people may be motivated to adopt protective behaviors not only to protect themselves but also to safeguard their social networks. Consistent with our findings, Barceló and Sheen observed during the COVID-19 pandemic in Spain that compliance with preventive behaviors, such as mask-wearing during COVID-19, was significantly influenced by shared expectations and prevailing social norms [34]. Similarly, it was found that among nurses in Korea, social support from peers and colleagues was the most significant predictor of adherence to health-protective behaviors [47]. These findings suggest that when people perceive their actions as contributing to the well-being of their group and receive appropriate support, they are more likely to engage in desired healthy behaviors. In informal settlements, where communal living, interdependence, and close social ties are integral to daily life, social motivation becomes a critical driver of behavior. Therefore, interventions that emphasize communal benefits of prevention and engage local leaders, community-based organizations, and peer networks may be needed to enhance these dynamics by reinforcing social norms and providing visible societal role models in informal urban settings.

Furthermore, the results indicate that prevention information did not show a significant direct relationship with prevention behaviors. This finding diverges from the hypothesized IMB skills model of pandemic risk and prevention [9] and contrasts with empirical studies that identified a significant relationship between information and behaviors [20]. However, this apparent contradiction is not entirely surprising when contextualized within the lived realities of marginalized communities. As evidenced by this study, the majority of participants (approximately 70%) were unable to answer all COVID-19–related knowledge questions correctly. This reveals not only a concerning COVID-19 knowledge gap but also a potentially deeper issue of disconnection between the dissemination of public health information and its reception, comprehension, and application at the community level. This may stem from misinformation or misconceptions, low health literacy, and inaccessible communication channels in low-resource settings, which have been well documented to obstruct the delivery and accurate interpretation of relevant public health messages, thereby influencing the engagement with recommended health behaviors [48, 49]. For instance, misconceptions fuel skepticism and erode trust in the uptake of pandemic prevention practices [49].

Significantly, the study’s finding that behavioral skills fully mediate the relationship between information and prevention behaviors reinforces the core principle of the IMB skills model. Our finding is consistent with that of [20, 21]. It suggests that, while important, the mere delivery and possession of information may not be sufficient to promote behavior change unless individuals are also equipped with practical, actionable skills. Indeed, as [9] argue that pandemic prevention information becomes impactful when accompanied by essential skills that boost self-efficacy and confidence, such as correct mask use, accurate symptom monitoring, and proper handwashing. While the study reveals the pivotal roles of skills, confidence, and relevant resources in informal urban settlements, these behavioral skills do not exist in isolation. Structural challenges, such as limited access to water, sanitation, and hygiene (WASH) facilities, overcrowding, and socio-economic conditions in informal urban settlements, often make the practicality of health behaviors challenging [2, 50, 51]. For example, crowded living conditions make social and physical distancing practically difficult even if residents have the relevant behavioral skills [2]. Similarly, even when individuals are trained in effective, regular handwashing, limited access to potable water or reliance on communal water sources (e.g., shared water points) means that even the most informed and skilled people must secure these basic needs amid high-risk pandemic interactions [52]. Consequently, our findings suggest that while strengthening behavioral skills is essential, such interventions will remain limited in impact unless accompanied by structural improvements and infrastructure provision, such as upgraded housing, clean water, sanitation, and relevant targeted safety nets/support. Accordingly, pandemic response strategies should integrate context-specific skill-building into awareness efforts, utilizing trusted community structures, culturally sensitive materials, and accessible, low-cost training for marginalized populations.

## Limitations of the Study

Despite its theoretical and empirical contributions, this study has some limitations. Firstly, the study focuses on three informal settlements in Ghana, which may limit the extent to which the findings can be generalized. However, structural vulnerabilities, such as overcrowding, high population density, growing socio-economic inequalities, and limited access to water, sanitation, and hygiene (WASH) services, are common across many informal settlements, especially in sub-Saharan Africa. As a result, the behavioral insights from this study, particularly the influence of behavioral skills and motivation (personal and social), can still help inform targeted pandemic risk communication and community health interventions in similar settings.

Secondly, while self-reported measures are established in behavioral scholarship, they remain susceptible to recall and social desirability biases [53]. This is particularly relevant here, as participants reflected on pandemic behaviors after restrictions had relaxed, increasing the risk of memory loss or post-facto rationalization. Although objective measures are generally more reliable [54], recall bias was minimized through the use of multiple reference points and timeline-based interview questions keyed to significant events (e.g., lockdowns). Future research should consider triangulating self-reports with direct observation to further enhance accuracy.

Thirdly, the pandemic prevention information construct was treated as an objectively observed, ordinal categorical variable. Thus, categorizing the variable into levels of functional mastery (the tipping point at which an individual has enough accurate information to feel confident engaging in behaviors) using Bloom’s cut-offs may reduce the granularity of statistical variance. However, this approach was prioritized to reflect community health literacy landscape in informal settlements, where reaching a cognitive threshold (e.g., the ability to effectively filter out misinformation) is more often essential. To account for the use of categorical data, we employed the WLSMV estimator [36], ensuring a robust analysis that balances statistical rigor with the practical reality of how health knowledge drives behavior in the community context.

Lastly, this study did not include several potentially influential variables, such as socio-demographic characteristics (e.g., gender, age, household size, educational attainment), as moderators. Indeed, past empirical studies have shown that these factors influence behaviors and may differ across genders, age, and income groups [55–57]. Future studies could investigate how these factors influence information, motivation (both personal and social), behavioral skills, and behavioral pathways, thereby helping untangle subgroup-specific dynamics in informal settlements. This enhanced understanding can support the creation of targeted policies to effectively tackle informational barriers, address the needs, motivations, and relevant skills development, and consider the circumstances of different groups in informal urban settlements.

## Conclusion

This study employed the SEM technique to investigate the complex factors influencing COVID-19 preventive behaviors, drawing on the IMB skills model of pandemic risk and prevention, and survey data from Ghana’s informal urban settlements. Results revealed a significant knowledge gap and demonstrated that information alone did not directly influence behavior. Instead, information influences behavioral skills, which in turn translate into the uptake of COVID-19 prevention behaviors. This emphasizes the need to go beyond information dissemination and to ensure skills development, self-efficacy, and the confidence necessary for health behavior change. The study also exemplifies the critical role of message framing—emphasizing that risk communication must be timely, factually accurate, emotionally engaging, socially relevant, and culturally appropriate. It is worth noting that these insights are not only crucial for understanding COVID-19 behavioral dynamics but also offer an invaluable evidence-based framework for shaping future pandemic preparedness and response strategies in low-resource settings.

## Supplementary Information

Below is the link to the electronic supplementary material.ESM 1(DOCX.19.5 KB)

## Data Availability

The data supporting the findings of this study are not publicly available due to ethical and privacy considerations involving human participants from vulnerable informal settlements. However, datasets may be made available upon reasonable request to the corresponding author, subject to approval by the relevant ethics committee and in compliance with data protection regulations.
