# Supplementary material for: Beyond COVID-19: Behavioral Drivers of Pandemic Prevention in Ghana’s Informal Urban Settlements
Source: J Urban Health. 2026 Jul 1;103(4):711–24. doi: 10.1007/s11524-026-01106-x (PMC13525133; doi:10.1007/s11524-026-01106-x)
Supplement: Supplementary file 1 [file 11524_2026_1106_MOESM1_ESM.docx]

**Table S1. Measurement items, response coding, descriptive statistics, and internal consistency of study constructs (N = 496)**

| **Construct** | **Measurement Items** | **Correct**  **Response** | | **Cronbach’s Alpha** |
| --- | --- | --- | --- | --- |
| **Pandemic Prevention Information (PPI)** | **PPI_1** = Could drinking unclean water make one contract COVID-19? | No | | 0.79 |
|  | **PPI_2** = Are the primary transmission methods of COVID-19 by droplets and contact with an infected person? | Yes | |  |
|  | **PPI_3** = Could drinking warm water, ginger, and lemon kill the COVID-19 virus? | No | |  |
|  | **PPI_4** = In general, once infected with COVID-19, does it take 2-14 days for symptoms to show? | Yes | |  |
|  | **PPI_5** = Could people with COVID-19 with no symptoms spread the virus to others? | Yes | |  |
|  | **PPI_6** = Could taking antibiotics prevent COVID-19? | No | |  |
|  | **PPI_7** = Is loss of smell or taste a symptom of COVID-19? | Yes | |  |
|  | **PPI_8** = Is the elderly the only group at risk of getting COVID-19? | No | |  |
|  | **PPI_9** = Could frequent handwashing prevent an individual from contracting COVID-19? | Yes | |  |
|  | **PPI_10** = Could a nose/face mask protect one from contracting COVID-19? | Yes | |  |
|  | | **Mean** | **SD** |  |
| **Pandemic Prevention Personal Motivation (PPPM)** | **PPPM_1** = I thought COVID-19 was very contagious | 4.07 | 0.85 | 0.90 |
|  | **PPPM_2** = I was afraid of contracting COVID-19 in 2020 because I could die | 4.04 | 0.89 |  |
|  | **PPPM_3** = I know that getting sick with COVID-19 can be very severe | 4.05 | 0.86 |  |
|  | **PPPM_4** = I believe that taking the recommended preventive measures can significantly reduce my risk of COVID-19 infection | 4.04 | 0.90 |  |
| **Pandemic Prevention Social Motivation (PPSM)** | **PPSM_1** = Following the appropriate recommended practices (e.g., a nose/face mask, washing of hands, applying hand sanitiser, etc.) could prevent those around me (family, friends and neighbours) from contracting COVID-19 | 3.89 | 0.91 | 0.83 |
|  | **PPSM_2** = My family and neighbours were willing to support my health behaviours. | 3.99 | 0.92 |  |
|  | **PPSM_3** = I believe that it is a shared responsibility within the community to collectively work toward preventing the spread of COVID-19 | 4.04 | 0.90 |  |
| **Pandemic Prevention Behavioural Skills (PPBS)** | **PPBS_1** = I actively paid attention to real-time COVID-19 information | 3.99 | 0.88 | 0.86 |
|  | **PPBS_2** = I could accurately identify and monitor my health for the common symptoms of COVID-19 | 3.92 | 0.90 |  |
|  | **PPBS_3** = I felt confident in my ability to protect myself against COVID-19 | 4.09 | 0.87 |  |
|  | **PPBS_4** = I could put on a face mask correctly, ensuring it covers my nose and mouth. | 4.03 | 0.85 |  |
| **Pandemic Prevention Behaviours (PPBH)** | **PPBH_1** = Wash hands regularly with soap and running water | 3.6 | 1.04 | 0.85 |
|  | **PPBH_2** = Apply alcohol-based hand sanitisers | 3.57 | 1.01 |  |
|  | **PPBH_3** = Maintain a safe physical distance from people when outside (at least 1 metre) | 3.07 | 1.18 |  |
|  | **PPBH_4** = Wear nose/face masks whenever I am in public places | 3.69 | 0.96 |  |
|  | **PPBH_5** = Cover my mouth and nose with a tissue or elbow when coughing or sneezing | 3.52 | 1.02 |  |
|  | **PPBH_6** = Avoid crowded areas | 3.45 | 1.15 |  |

*Notes: M = mean; S.D = standard deviation*
